# Evolution of Perioperative Outcomes in Robot-Assisted Radical Cystectomy over 20 Years of Experience in a High-Volume Tertiary Robotic Center

**DOI:** 10.3390/cancers17183060

**Published:** 2025-09-19

**Authors:** Simone Morra, Stefano Resca, Nicola Frego, Sara Tamburini, Marco Ticonosco, Alessandro Pissavini, Andrea Noya Mourullo, Francesco Barletta, Mario de Angelis, Edward Lambert, Frederiek D’Hondt, Ruben De Groote, Geert De Naeyer, Alexandre Mottrie

**Affiliations:** 1 Department of Urology, AZORG Hospital, 9300 Aalst, Belgium; stefano.resca95@gmail.com (S.R.); nicola.frego@gmail.com (N.F.); sara.tamburini3@studio.unibo.it (S.T.); marco.ti151992@gmail.com (M.T.); alessandro.pissavini@studio.unibo.it (A.P.); barletta.francesco@hsr.it (F.B.); deangelis.mario@hsr.it (M.d.A.); edward.lambert@olvz-aalst.be (E.L.); frederiek.dhondt@olvz-aalst.be (F.D.); degroote.ruben@gmail.com (R.D.G.); geert.de.naeyer@olvz-aalst.be (G.D.N.); a.mottrie@gmail.com (A.M.); 2 ORSI Academy, 9090 Melle, Belgium; 3 Department of Neurosciences, Reproductive Sciences and Odontostomatology, School of Medicine, University of Naples “Federico II”, 80131Naples, Italy; 4 Department of Urology, University of Modena and Reggio Emilia, 41121 Modena, Italy; 5 Department of Urology, Humanitas Research Hospital, IRCCS, 20089 Milan, Italy; 6 Division of Urology, IRCCS Azienda Ospedaliero, Universitaria di Bologna, 40138 Bologna, Italy; 7 Urology Department, University Hospital of Salamanca, 37007 Salamanca, Spain; 8 UROINTEC Urologia, Innovación, Tecnología, 35001 Las Palmas de Gran Canaria, Spain; 9 Division of Oncology, Unit of Urology, URI, IRCCS Ospedale San Raffaele, 20132 Milan, Italy

**Keywords:** robot-assisted radical cystectomy, perioperative outcomes, trend analysis, 20 years’ experience

## Abstract

Background/Objectives: Robot-assisted radical cystectomy (RARC) has demonstrated improved perioperative outcomes and recovery in bladder cancer (BCa) patients. This study compares patient and tumor characteristics, operative time (OT), length of stay (LOS), and complication rates between a historical (2003–2016) and a contemporary cohort (2017–2024) treated at a high-volume robotic center. Methods: Data from 274 BCa patients who underwent RARC at AZORG Hospital, Aalst, Belgium, were analyzed. Perioperative outcomes were compared between cohorts. Multivariable Poisson regression models identified predictors of longer OT and LOS, while multivariable logistic regression models (MLRMs) assessed predictors of higher complication rates. Results: Overall, 274 BCa patients who underwent RARC were identified (38% historical cohort vs. 62% contemporary cohort). The contemporary cohort had a significantly shorter median OT (345 vs. 360 min; *p* = 0.048) and LOS (8 vs. 12 days; *p* < 0.001) compared to the historical cohort. Postoperative complications were lower in the contemporary group, with more cases experiencing no complications (60% vs. 41%) and fewer grade 3–4 complications (10% vs. 27%; *p* < 0.001). In multivariable Poisson regression, the contemporary cohort was an independent predictor of shorter OT (Incidence Rate Ratio [IRR]: 0.94, 95% [Confidence Interval] CI: 0.93–0.96; *p* = 0.04) and shorter LOS (IRR: 0.65, 95% CI: 0.60–0.69; *p* < 0.001). In MLRMs predicting complications, the contemporary cohort was associated with lower risk (Odds Ratio: 0.42, 95% CI: 0.23–0.76; *p* = 0.005). Conclusions: RARC outcomes improved significantly over time, with reduced OT, LOS, and complication rates in the contemporary cohort, highlighting advancements in surgical techniques, perioperative care, and patient safety. These findings reinforce the role of RARC in optimizing BCa treatment.

## Simple Summary

Radical cystectomy (RC) is a technically demanding procedure, traditionally associated with considerable postoperative morbidity and prolonged recovery. The adoption of robot-assisted radical cystectomy (RARC) has introduced opportunities for greater surgical precision, enhanced perioperative management, and potentially improved patient outcomes. This study investigates temporal changes in the results of this procedure within a high-volume robotic surgery center. By comparing outcomes from an earlier era to those from a more recent period, the authors aim to determine whether advances in surgical technique, perioperative protocols, and patient care have translated into measurable benefits. Key metrics assessed include operative time, hospital length of stay, and the incidence and severity of postoperative complications. The findings demonstrate significant improvements over time, underscoring the impact of continuous refinement in surgical practice. These results may inform clinical decision-making, support the optimization of treatment pathways, and contribute to establishing updated benchmarks for bladder cancer surgery in specialized centers.

## 1. Introduction

Radical cystectomy (RC) is the gold standard treatment for patients with muscle-invasive and recurrent high-risk non–muscle-invasive bladder cancer (BCa) [1]. Despite the increasing use of trimodal therapy, radical cystectomy remains a standard curative treatment, involving the removal of the bladder and the surrounding lymph nodes [2,3]. Despite its efficacy, RC is associated with significant morbidity, making the optimization of surgical techniques crucial for improving patient outcomes [4]. Indeed, the peri-operative mortality after RC was reported as 2.1–3.2% at 30 days and 3.4–8.0% at 90 days [5,6]. Interestingly, lower morbidity was recorded in hospitals with a higher caseload, indicating greater experience [7]. For example, a recent work by Liedeberg et al. reported significant reductions in 90-day mortality and re-operation rates in 4638 RC patients from the Swedish national database due to the centralization of RC services from 24 centers to ten [8].

In recent years, robotic-assisted RC (RARC) has emerged as a minimally invasive alternative to open surgery, offering potential benefits such as reduced blood loss, shorter hospitalization, and faster recovery [9]. For instance, Tamhankar et al. recorded a statistically significant increase in the number of RARC from 10.8% in 2013–2014 to 39.5% in 2018–2019 in England [10]. Several research groups have explored these advantages, highlighting improved perioperative outcomes and reduced complications with robotic techniques compared to traditional approaches [11]. However, the adoption of RARC has also prompted discussions on its learning curve, operative times, cost-effectiveness and long-term oncological outcomes [12,13].

Our study aims to assess the evolution of the outcomes of patients who underwent RARC over 20-years of experience at a high-volume robotic referral center. The objective is to identify predictors of worse perioperative outcomes, such as prolonged operative time (OT), prolonged length of stay (LOS), and higher complication rates. Additionally, we perform a comparative analysis of two cohorts—one representing an earlier period (2003–2016) and the other a more contemporary group (2017–2024)—to evaluate temporal trends and improvements in outcomes associated with RARC.

## 2. Materials and Methods

### 2.1. Study Population

**Figure 1:**
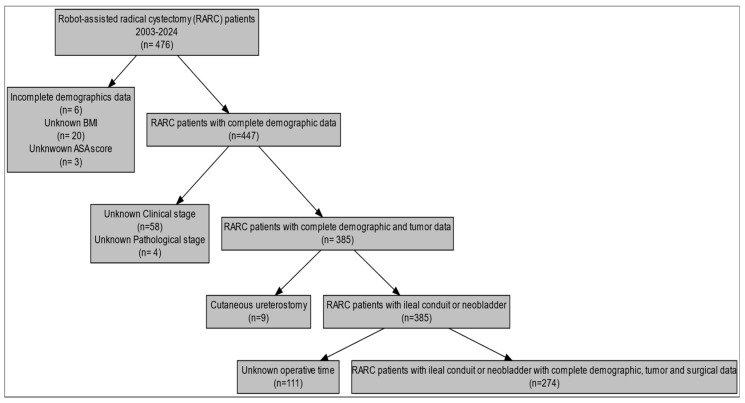
Flowchart of selection of RARC patients.

The current study relied on the AZORG Hospital (Aalst, Belgium) prospectively maintained institutional database (ethical committee approval number: 2014/055). Specifically, all patients aged ≥18 yr who underwent RARC for histologically confirmed BCa at AZORG Hospital between July 2003 and March 2024 were identified. Only patients with complete baseline, preoperative, perioperative, postoperative, and pathological data were included. Due to very few cases (*n* = 10), we excluded patients who underwent RARC with cutaneous ureterostomy (Figure 1). All surgeries were performed by three (A.M, F.D, E.L) experienced surgeons (more than 150 robot-assisted procedures performed). The robotic systems used were the DaVinci^®^ Si, X, and Xi (Intuitive Surgical, California, FL, USA). All the procedures were performed using the following setting: four robotic arms (0° endoscope, monopolar curved scissors (coagulation and cut set at three), bipolar Maryland (coagulation set at four), large needle driver, and Prograsp forceps.

### 2.2. Variables and Outcome of Interest

For each patient, the following variables of interest were recorded: age at surgery (years), sex, body mass index (BMI) (kg/m^2^, continuously coded), American Society of Anesthesiologists (ASA) score (continuously coded), Charlson Comorbidity Index (CCI) (<4 vs. ≥4), smoking status (current vs. former vs. never vs. NA), previous abdominal surgery (yes vs. no), clinical stage (cTa-isN0M0 vs. cT1N0M0 vs. cT2N0M0 vs. cT3-4N0M0 vs. cTanyN + M0), carcinoma in situ at TURB (yes vs. no), operative time (OT) (minutes, continuously coded), length of stay (LOS) (days, continuously coded), complications according to Clavien–Dindo classification (No complications vs. Grade 1–2 vs. Grade 3–4), lymph node dissection (not performed vs. standard vs. extended), pathological T stage at RARC (pT0 vs. pT1 vs. pT2 vs. pT3 vs. pT4), pathological N stage (pN+ vs. pN0 vs. pNx), and urinary diversion type (UD) (Ileal conduit vs. neobladder).

### 2.3. Statistical Analyses

Four analytical steps were completed. First, we tabulated baseline patients and tumor clinical characteristics. Second, we tabulated pathological features and perioperative outcomes of all patients. All the tables were stratified according to the year of surgery (2003–2016 vs. 2017–2024). Descriptive statistics included frequencies and proportions for categorical variables. Medians and interquartile ranges (IQR) were reported for continuously coded variables. The Kruskal–Wallis and the chi-squared tests were used to compare medians and proportions, respectively. Third, multivariable Poisson linear regression models were fitted to identify independent predictors of longer OT and LOS. Fourth, a multivariable logistic regression model (MLRM) was fitted to identify independent predictors of higher complication rates of any grade according to Clavien–Dindo classification (no vs. any grade). In all multivariable logistic regression analyses, the number of covariates met the criteria for model overfitting prevention. All tests were two-sided, with a significance level set at *p* < 0.05. In all statistical analyses, the R software environment for statistical computing and graphics (R version 4.1.3, R Foundation for Statical Computing, Vienna Austria; http://www.r-project.org/, accessed on 5 April 2024) was used [14].

## 3. Results

### 3.1. Descriptive Characteristics

Overall, 274 BCa patients who underwent RARC between July 2003 and March 2024 were identified. Descriptive characteristics are reported in Table 1. Specifically, 38% (*n* = 103) harbored the historical cohort (2003–2016) vs. 62% (*n* = 171) the contemporary one (2017–2024). According to the year of surgery, the contemporary patients were statistically significantly older (72 vs. 69 years; *p* = 0.04), more frequently former smokers (44% vs. 34%), and less frequently never-smokers (16% vs. 33%; *p* = 0.01) relative to their historical counterparts. Moreover, the contemporary group more frequently harbored CCI ≥4 (85% vs. 55%; *p* < 001), and was more frequently exposed to neoadjuvant chemotherapy (39% vs. 27%; *p* = 0.04), compared to their historical counterparts. No differences were recorded in BMI (*p* = 0.7), sex (*p* = 0.08), ASA score (*p* = 0.06), CCI (*p* = 0.3), and previous abdominal surgery (*p* = 0.2). Regarding tumor characteristics, carcinoma in situ rates (13% vs. 25%; *p* = 0.01) were lower in the contemporary cohort relative to the historical one. No statistically significant difference was recorded in clinical stage (*p* = 0.9).

### 3.2. Pathological Features and Perioperative Outcomes

The median OT of all RARCs was 350 min (IQR: 300–420). After stratification by the year of surgery, a statistically significant reduction in the median OT was observed in the contemporary cohort compared to the historical cohort (345 vs. 360 min; *p* = 0.048). Similarly, the median LOS was 10 days (IQR: 7–14), with a statistically significant decrease in the contemporary cohort compared to the historical counterpart (8 vs. 12 days; *p* < 0.001) (Table 2).

Postoperative complication rates also differed significantly between the two cohorts. Specifically, the contemporary cohort showed a higher percentage of cases with no complications (60% vs. 41%), fewer cases with Clavien–Dindo grade 1–2 complications (30% vs. 32%), and fewer cases with Clavien–Dindo grade 3–4 complications (10% vs. 27%; *p* < 0.001) (Table 2).

No statistically significant differences were noted between the cohorts in terms of pathological T stage (*p* = 0.13), pathological N stage (*p* = 0.4), and urinary diversion type (*p* = 0.2). However, differences in lymph node dissection as well as in carcinoma in situ rates were recorded. Specifically, extended lymph node dissection was less frequently performed in the contemporary cohort compared to the historical one (39% vs. 65%; *p* < 0.001), and carcinoma in situ rate was lower in the contemporary cohort compared to the historical one (17% vs. 30%; *p* = 0.01) (Table 2).

### 3.3. Multivariable Regression Models

In the multivariable Poisson linear regression model predicting longer OT, harboring the contemporary cohort (2017–2024) was an independent predictor of shorter OT (Incidence Rate Ratio [IRR]: 0.94, 95% Confidence Interval [CI]: 0.93–0.96; *p* = 0.04), as well as older age (IRR: 0.99, *p* < 0.001), and harboring CCI ≥ 4 (IRR: 0.98, *p* = 0.002). Conversely, the neobladder urinary diversion independently predicted longer OT (IRR: 1.17, *p* < 0.001) as well as higher BMI (IRR: 1.004, *p* < 0.001) (Table 3). In the multivariable Poisson linear regression model predicting longer LOS, harboring the contemporary cohort was an independent predictor of shorter LOS (IRR: 0.65, 95% CI: 0.60–0.69, *p* < 0.001). Conversely, older age (IRR: 1.01, *p* < 0.001), higher BMI (IRR:1.009, *p* = 0.03), neobladder urinary diversion (IRR:1.40, *p* < 0.001) were associated with longer LOS (Table 4). In MLRMs predicting postoperative complication of any grade according to Clavien–Dindo classification, harboring the contemporary cohort was an independent predictor of lower complication rates (OR: 0.42, 95% CI: 0.23–0.76; *p* = 0.005) (Table 5).

## 4. Discussion

To date, RARC has emerged as a minimally invasive alternative to open surgery, offering potential benefits such as reduced blood loss, shorter hospital stay, and faster recovery [9]. However, challenges persist, particularly regarding the learning curve required to optimize outcomes. Moreover, the EAU guidelines increasingly emphasize the importance of referring patients to referral high-volume centers [15]. These centers, benefiting from greater surgical experience due to higher case volumes, have reported lower complication rates [7].

Our study aims to describe the evolution of baseline patient characteristics and tumor features treated with RARC at our tertiary referral center. Additionally, we seek to highlight potential differences between patients treated in an earlier cohort (2003–2016) and those treated in a more recent cohort (2017–2024). Furthermore, our work aims to identify potential predictors of unfavorable perioperative outcomes, such as prolonged LOS and OT, as well as predictors of complications of any grade according to the Clavien–Dindo classification. Our analyses highlighted several noteworthy observations.

First, our study encompasses a substantial cohort of 274 patients who underwent RARC with intracorporeal UD at a single tertiary care center. The comprehensive nature and high quality of our data render this cohort representative of the general population undergoing such procedures. Comparable studies from other tertiary care centers have reported similar patient volumes, underscoring the relevance of our findings. Similarly, Tae et al. focused on oncological and functional outcomes during the learning curve of RARC, collecting throughout 11 years, 120 BCa patients treated with RARC at a single tertiary hospital [16]. Interestingly, they recorded that RARC outcomes, both oncological and functional, were comparable to open radical cystectomy [16].

Second, our analysis revealed statistically significant differences in patient characteristics between the historical (2003–2016) and contemporary (2017–2024) cohorts, notably in age. The median age at cystectomy increased from 69 years in the earlier group to 72 years in the latter, indicating that minimally invasive robotic surgery, specifically RARC, has become a viable option for an increasingly older patient population over time. This trend aligns with findings from other studies demonstrating the safety and feasibility of RARC in elderly patients. Specifically, Yu et al. focusing on the impact of RC in perioperative mortality in octogenarian patients, recorded lower in-hospital mortality in RARC patients compared to their open surgery counterparts, suggesting that elderly patients may derive more benefit from minimally invasive RC compared to a younger cohort [17]. Additionally, Tanabe et al. have reported that the incidence of perioperative complications in patients aged 80 years or older who underwent RARC is comparable to that in non-elderly patients, further supporting the procedure’s applicability in older populations [18].

Third, our results revealed a statistically significant reduction in median OT in the contemporary cohort compared to the historical cohort (345 vs. 360 min; *p* = 0.048). This suggests an improvement in surgical efficiency over time. Moreover, in the multivariable Poisson regression model predicting longer OT, belonging to the contemporary cohort (2017–2024) was an independent predictor of shorter OT (IRR: 0.94, 95% CI: 0.93–0.96; *p* = 0.04), indicating that advancements in surgical techniques, perioperative management, or patient selection criteria may have contributed to this trend. Further studies are warranted to explore the specific factors driving this improvement. This finding aligns with previous studies indicating that the complexity of neobladder reconstruction contributes to longer surgical durations. Specifically, Yasuda et al., analyzing surgical outcomes of RARC, reported that prolonged OT was a significant prognostic factor for major complications, emphasizing the impact of complex urinary diversions on surgical time [19]. An additional factor that may have contributed to the improved outcomes observed in the contemporary cohort is the evolution of robotic surgical platforms, transitioning from the Da Vinci Si to the newer X and Xi systems [20]. The Xi platform introduced several technical refinements, including overhead boom architecture, slimmer arms with increased range of motion, and more efficient docking, all of which enhance surgical ergonomics and reduce operative time variability [21]. These technological advancements have been associated with improved perioperative outcomes in complex urological procedures, such as robot-assisted radical prostatectomy and cystectomy, potentially explaining the reduction in OT and LOS observed in our cohort. Future studies directly comparing outcomes between different robotic platforms in RARC could further clarify the extent to which these technological advances impact perioperative metrics.

Fourth, we recorded a median LOS of 10 days in the overall cohort. These results are in line with previous published papers. Indeed, Lee et al. reported in a large Asian series of RARC (2007–2020) a median LOS of 13 days [22]. Our faster discharge results are mostly due to the inclusion of more contemporary years as well as a different healthcare system. Indeed, Johar et al. recorded a median LOS of 8 days from the International Robotic Cystectomy Consortium database, an international, multi-institutional database, predominantly composed of European countries, with additional participating centers from North America, Asia, and the Middle East [23]. We recorded a statistically significant reduction in median length of stay (LOS) in the contemporary cohort compared to the historical cohort (8 vs. 12 days; *p* < 0.001) was recorded. Moreover, in the multivariable Poisson linear regression model predicting longer LOS, harboring the contemporary cohort was an independent predictor of shorter LOS (IRR: 0.65, 95% CI: 0.60–0.69, *p* < 0.001). These findings align with recent studies indicating that RARC is associated with shorter hospital stays. For instance, a Japanese nationwide database study reported that minimally invasive radical cystectomies had a significantly shorter LOS compared to open procedures [24]. Checking for predictors of prolonged LOS (LOS ≥ 10), Polverino et al. (National Inpatient Sample [NIS] 2006–2019)recorded that the chronic liver disease (CLD) was an independent predictor of prolonged LOS (OR: 1.4, *p* = 0.02) [25]. Unfortunately, we can only rely on the CCI as a proxy of CLD and no statistically significant correlation was associated with the LOS. Regarding perioperative complications, we recorded no complication in the majority of the patients (53%) and very few Clavien–Dindo ≥3 complications (16%). This data is similar to the large Asian RARC series reported by Lee et al. (15.6%) [22]. Interestingly, the contemporary cohort exhibited a higher percentage of cases with no complications (64% vs. 42%) and fewer cases with Clavien–Dindo grade 3–4 complications (10% vs. 27%; *p* < 0.001). Multivariable analysis confirmed that being part of the contemporary cohort was an independent predictor of lower complication rates (OR: 0.43, 95% CI: 0.24–0.73; *p* = 0.002). This improvement in outcomes is consistent with findings from other studies. For example, a systematic review reported that RARC was associated with lower estimated blood loss, lower intraoperative transfusion rates, shorter LOS, and a lower risk of complications compared to open radical cystectomy [26]. Notably, the BMI was recorded as an independent predictor by Lee et al. for complication of any grade in RARC patients (OR: 1.65, *p* = 0.018) as well as by Johar et al. (OR: 1.04, *p* = 0.006), but within our population, BMI was not an independent predictor (OR: 0.99, *p* = 0.8) [22,23]. These enhancements in LOS and perioperative complications may be attributed to advancements in surgical techniques, increased surgeon experience, and improved perioperative care protocols over time. The adoption of minimally invasive approaches like RARC has played a significant role in enhancing patient recovery and reducing the incidence of complications.

Fifth, our results showed a difference in terms of LND patterns as well as in the rate of pT0/pT_a-is_ rates between the contemporary vs. the historical cohort. Interestingly, we observed a statistically significant difference in Extended LND that was more frequently performed in the historical cohort (65% vs. 39%), whereas a higher proportion of standard-template dissections was observed in the contemporary cohort (42% vs. 16%). The rate of non-performed LND remained unchanged between groups (19% vs. 19%). This shift likely reflects evolving surgical decision-making and increased surgeon confidence in patient selection and disease management. Additionally, we observed a higher, although not statistically significant, proportion of pT0 and pTa/pTis cases in the contemporary cohort compared with the historical cohort (pT0: 31% vs. 17%; pTa/pTis: 17% vs. 14%). This trend may be attributed to the increased use in our center of neoadjuvant chemotherapy over time (27% vs. 39%, *p* = 0.04), which has been consistently associated with higher pT0 rates at radical cystectomy in previous reports [27]. In support of this interpretation, Peyton et al. reported that among 824 patients with muscle-invasive bladder cancer, neoadjuvant chemotherapy was associated with significantly higher rates of pathologic complete response: for example, 41.3% for a dose-dense combination of methotrexate, vinblastine, doxorubicin, and cisplatin (ddMVAC) and 24.5% for gemcitabine-cisplatin [28]. Thus, our observed trend toward higher pT_0_/pT_a-is_ in the contemporary cohort, although not reaching statistical significance, is consistent with published data and may indeed reflect the increasing adoption of more intensive neoadjuvant chemotherapy regimens. Ultimately, these changes underscore the importance of adjusting for both LND extent and pathologic stage when interpreting temporal shifts in outcomes.

Taken together, our study focused on RARC, a procedure increasingly adopted worldwide as a cornerstone in the management of muscle-invasive BCa. Our findings highlight the expanding applicability of minimally invasive approaches such as RARC, particularly in older patients. This is evidenced by the median age in the contemporary cohort, which was significantly higher at 72 years compared to 69 years in the historical cohort. Moreover, when comparing the contemporary cohort to the historical group, we observed significant improvements in key perioperative outcomes. These include a reduction in operative time—though this was not confirmed by multivariate analysis—a statistically significant decrease in length of stay, and, most importantly, a marked reduction in perioperative complications. The latter reflects both increased surgical expertise and the implementation of improved perioperative care protocols.

These findings underscore the growing role of RARC as a safe, effective, and increasingly versatile option for managing bladder cancer, particularly as surgical techniques and perioperative strategies continue to evolve.

Our study is not devoid of limitations. First and foremost, this study is retrospective in nature, relying on data from a prospectively maintained database, which may introduce inherent biases despite the robustness of the dataset. The lack of randomization limits the ability to establish causation between cohort differences and outcomes. Second, the study lacks granular data on surgeon learning curves, the potential influence of temporal changes in anesthetic and peri-operative care, surgical technique, and technology since we relied on a wide timeframe of 21 years. Specifically, the introduction of ERAS protocols in 2013 [29], refinement of lymph node dissection techniques, and the shift towards intracorporeal reconstruction [30] are likely to have improved peri-operative outcomes and may partially account for differences observed between cohorts [31]. These factors likely contributed to the improved outcomes in the contemporary cohort but were not explicitly analyzed, limiting the ability to identify which changes had the most impact. Moreover, the higher proportion of pT0 cases and the absence of lymph node dissection in a subset of contemporary patients may have influenced oncological outcomes and should be interpreted with caution. These factors highlight the complexity of comparing results across different time frames and underscore the need for future prospective studies accounting for these variables. Third, while the sample size of 274 patients is substantial and representative of a high-volume tertiary center, the findings may not be generalizable to lower-volume centers or different healthcare systems, where surgical experience and perioperative care protocols may vary. Addressing these limitations in future prospective, multicenter studies will provide deeper insights and broader applicability of the findings.

## 5. Conclusions

RARC outcomes improved significantly over time, with shorter OT, reduced LOS, and lower complication rates in the contemporary cohort, reflecting advancements in surgical efficiency, postoperative care, and safety. These findings underscore the evolving benefits of RARC in optimizing BCa treatment.

## Figures and Tables

**Table 1:** Baseline demographics and Clinical Characteristics of 274 patients who underwent robot-assisted radical cystectomy (RARC) at AZORG Hospital between 2003 and 2024.

Characteristic	Overall *n* = 274 ^1^	Historical Group 2003–2016, *n* = 103 (38%) ^1^	Contemporary Group 2017–2024, *n* = 171 (62%) ^1^	*p*-Value ^2^
**Age** (years)	71 (63, 77)	69 (62, 76)	72 (64, 78)	0.04
**BMI** (kg/m^2^)	26.5 (23.5, 28.7)	26.5 (23.0, 28.6)	26.5 (23.6, 29.4)	0.7
**Charlson comorbidity index**				<0.001
<4	72 (26%)	46 (45%)	26 (15%)	
≥4	202 (74%)	57 (55%)	145 (85%)	
**Males**	225 (82%)	90 (87%)	135 (79%)	0.08
**ASA score**				0.06
1	14 (5%)	5 (5%)	9 (55%)	
2	184 (67%)	61 (59%)	123 (72%)	
3	76 (28%)	37 (36%)	39 (23%)	
**Smoking status**				0.005
Current	54 (20%)	22 (21%)	32 (19%)	
Former	110 (40%)	35 (34%)	75 (44%)	
Never	62 (23%)	34 (33%)	28 (16%)	
NA	48 (18%)	12 (12%)	36 (21%)	
**Clinical stage**				0.9
cT_a-is_N_0_M_0_	21 (8%)	10 (10%)	11 (6%)	
cT_1_N_0_M_0_	42 (15%)	15 (15%)	27 (16%)	
cT_2_N_0_M_0_	133 (49%)	49 (48%)	84 (49%)	
cT_3–4_N_0_M_0_	31 (11%)	12 (12%)	19 (11%)	
cT_any_N + M_0_	47 (17%)	17 (17%)	30 (18%)	
***Carcinoma*** **in situ**	49 (18%)	26 (25%)	23 (13%)	0.01
**Previous abdominal surgery**	89 (32%)	29 (28%)	60 (35%)	0.2
**Neoadjuvant chemotherapy**	95 (35%)	28 (27%)	67 (39%)	0.04

^1^ Median (Q1, Q3); *n* (%). ^2^ Wilcoxon rank sum test; Pearson’s Chi-squared test. Abbreviations: BMI = Body Mass Index, ASA = American Society of Anestesiology.

**Table 2:** Pathological features and perioperative outcomes of 278 patients who underwent robot-assisted radical cystectomy (RARC) at AZORG Hospital between 2003 and 2024.

Characteristic	Overall *n* = 274 ^1^	Historical 2003–2016, *n* = 103 (38%) ^1^	Contemporary 2017–2024, *n* = 171 (62%) ^1^	*p*-Value ^2^
**Operative time**	350 (300, 420)	360 (320, 420)	345 (300, 410)	0.048
**Length of stay**	10 (7, 14)	12 (9, 16)	8 (7, 12)	<0.001
**Clavien–Dindo complications**				<0.001
No complications	145 (53%)	42 (41%)	103 (60%)	
Grade 1–2	84 (31%)	33 (32%)	51 (30%)	
Grade 3–4	45 (16%)	28 (27%)	17 (10%)	
**Urinary diversion**				0.4
Ileal conduit	241 (88%)	93 (90%)	148 (87%)	
Neobladder	33 (12%)	10 (9.7%)	23 (13%)	
**Lymph node dissection**				<0.001
Not performed	52 (19%)	20 (19%)	32 (19%)	
Standard	89 (32%)	16 (16%)	73 (42%)	
Extended	133 (49%)	67 (65%)	66 (39%)	
**Pathological T stage**				0.2
pT0	71 (26%)	18 (17%)	53 (31%)	
pT_a-is_	42 (15%)	18 (17%)	24 (14%)	
pT1	31 (11%)	11 (11%)	20 (12%)	
pT2	47 (17%)	23 (22%)	24 (14%)	
pT3	65 (24%)	25 (24%)	40 (23%)	
pT4	18 (7%)	8 (8%)	10 (6%)	
**Pathological N stage**				0.4
pN+	37 (14%)	15 (15%)	22 (13%)	
pN0	200 (73%)	78 (76%)	122 (71%)	
pNx	37 (14%)	10 (9.7%)	27 (16%)	
***Carcinoma*** **in situ**	60 (22%)	31 (30%)	29 (17%)	0.01

^1^ Median (Q1, Q3); *n* (%). ^2^ Wilcoxon rank sum test; Pearson’s Chi-squared test.

**Table 3:** Multivariable Poisson linear regression model predicting longer operative time in patients who underwent robot-assisted radical cystectomy (RARC) between 2003 and 2024 at AZORG Hospital.

Characteristic	IRR	95% CI	*p*-Value
**Age**	0.99	0.994, 0.995	**<0.001**
**Body mass index**	1.004	1.003, 1.006	**<0.001**
**Year of surgery**			
*2003–* *2016*	—	—	
*2017–2024*	0.94	0.93, 0.96	**<0.001**
**Charlson comorbidity index**			
*<4*	—	—	
*>=4*	0.98	0.96, 0.99	**0.002**
**Previous abdominal surgery**			
*No*	—	—	
*Yes*	1.03	1.01, 1.04	**<0.001**
**Urinary diversion**			
*Ileal conduit*	—	—	
*Neobladder*	1.17	1.15, 1.19	**<0.001**

Abbreviations: CI = Confidence Interval, IRR = Incidence Rate Ratio.

**Table 4:** Multivariable Poisson linear regression model predicting longer Length of stay (LOS) in patients who underwent robot-assisted radical cystectomy (RARC) between 2003 and 2024 at AZORG Hospital.

Characteristic	IRR	95% CI	*p*-Value
**Age**	1.01	1.008, 1.017	**<0.001**
**Body mass index**	1.009	1.001, 1.02	**0.03**
**Year of surgery**			
*2003–2016*	—	—	
*2017–2024*	0.65	0.60, 0.69	**<0.001**
**Charlson comorbidity index**			
*<4*	—	—	
*>=4*	0.94	0.86, 1.02	0.1
**Urinary diversion**			
*Ileal conduit*	—	—	
*Neobladder*	1.40	1.26, 1.57	**<0.001**

Abbreviations: CI = Confidence Interval, IRR = Incidence Rate Ratio.

**Table 5:** Multivariable logistic regression models predicting post-operative complications of any grade (Clavien–Dindo > 0) in patients who underwent robot-assisted radical cystectomy (RARC) at AZORG Hospital between 2003 and 2024.

Characteristic	OR	95% CI	*p*-Value
**Age**	1.00	0.97, 1.04	0.8
**BMI**	0.99	0.94, 1.05	0.8
**Charlson comorbidity index**			
*<4*	—	—	
*>=4*	1.24	0.67, 2.31	0.5
**Year of surgery**			
*2003–2016*	—	—	
*2017–2024*	0.43	0.24, 0.73	**0.002**
**Previous abdominal surgery**			
*No*	—	—	
*Yes*	1.26	0.74, 2.13	0.4
**Neoadjuvant chemotherapy**			
*No*	—	—	
*Yes*	0.90	0.53, 1.52	0.7
**Urinary diversion**			
*Ileal conduit*	—	—	
*Neobladder*	0.712	0.30, 1.65	0.4

Abbreviations: CI = Confidence Interval, OR = Odds Ratio, BMI = Body Mass Index.

## Data Availability

The data supporting the findings of this study are available from the corresponding author upon reasonable request. Due to patient confidentiality and institutional regulations, restrictions apply to the availability of these data.
